# Bioassay-Guided Isolation of Sesquiterpene Coumarins from *Ferula narthex* Bioss: A New Anticancer Agent

**DOI:** 10.3389/fphar.2016.00026

**Published:** 2016-02-16

**Authors:** Mahboob Alam, Ajmal Khan, Abdul Wadood, Ayesha Khan, Shumaila Bashir, Akhtar Aman, Abdul Khaliq Jan, Abdur Rauf, Bashir Ahmad, Abdur Rahman Khan, Umar Farooq

**Affiliations:** ^1^Department of Pharmacy, Hazara UniversityDhodial, Pakistan; ^2^Department of Chemistry, COMSATS Institute of Information TechnologyAbbottabad, Pakistan; ^3^Department of Biochemistry, Abdul Wali Khan University-MardanMardan, Pakistan; ^4^Department of Pharmacy, University of PeshawarPeshawar, Pakistan; ^5^Department of Pharmacy, Shaheed Benazir Bhutto University SheringalSheringal, Pakistan; ^6^Centre for Phytomedicine and Medicinal Organic Chemistry, Institute of Chemical Sciences, University of PeshawarPeshawar, Pakistan; ^7^Centre of Biotechnology and Microbiology, University of PeshawarKhyber Pakhtunkhwa, Pakistan

**Keywords:** *Ferula narthex* Bioss, sesquiterpene coumarin, cytotoxic, anticancer potential, Prediction of Activity Spectra, human histone acetyltransferase, and molecular docking

## Abstract

The main objective of cancer management with chemotherapy (anticancer drugs) is to kill the neoplastic (cancerous) cell instead of a normal healthy cell. The bioassay-guided isolation of two new sesquiterpene coumarins (compounds **1** and **2**) have been carried out from *Ferula narthex* collected from Chitral, locally known as “Raw.” Anticancer activity of crude and all fractions have been carried out to prevent carcinogenesis by using MTT assay. The *n*-hexane fraction showed good activity with an IC_50_ value of 5.434 ± 0.249 μg/mL, followed by crude MeFn extract 7.317 ± 0.535 μg/mL, and CHCl_3_ fraction 9.613 ± 0.548 μg/mL. Compounds 1 and 2 were isolated from chloroform fraction. Among tested pure compounds, compound **1** showed good anticancer activity with IC_50_ value of 14.074 ± 0.414 μg/mL. PASS (Prediction of Activity Spectra) analysis of the compound **1** was carried out, in order to predicts their binding probability with anti-cancer target. As a results the compound **1** showed binding probability with human histone acetyltransferase with Pa (probability to be active) value of 0.303. The compound **1** was docked against human histone acetyltransferase (anti-cancer drug target) by using molecular docking simulations. Molecular docking results showed that compound **1** accommodate well in the anti-cancer drug target. Moreover the activity support cancer chemo preventive activity of different compounds isolated from the genus *Ferula*, in accordance with the previously reported anticancer activities of the genus.

## Introduction

*Ferula narthex* Bioss; family Apiaceae, is indigenous to Kandahar, Eastern Persia, Western Afghanistan and Pakistan (Kashmir and Baltistan; Indrayan et al., 2009). In Pakistan it is found in various localities like Gilgit, Chitral (Kamari, Damusar, Chilim, Gudai, Astore, and the hill of Majini Harai), locally it is known as “Raw” in Chitral (Shinwari and Gilani, 2003). Family Apiaceae (Umbelliferae) comprises of 275 genera and 2850 species (Indrayan et al., 2009). *F. narthex* Bioss. is a well-known carminative, anti-flatulence digestive spice, much essential for Ayurvedic formulation and even for the preparation of many Indian dishes i.e., Asafoetida botanically known as *Ferula narthex* Bioss (Sengupta et al., 2004). It is an oleogum-resin collected from rhizome and root of the plant. It is considered as very important agent in Indian system of medicine, especially for intestinal flatulence. It is acrid, bitter, stimulant and mostly useful in constipation-gaseous distention (colic pain; Sengupta et al., 2004). Local people used this plant for cough, asthama, toothache, gastric problems and in constipation, angina pectoris. Gum resin of *Ferula narthex* Bioss. is used in hysteria, treatment of habitual abortion, whooping cough and scorpion sting (Srinivasan, 2005; Anuar et al., 2008; Khan et al., 2011). Extracts and pure compounds from this plant showed anticancer (Saleem et al., 2001), antidiabetic (Iranshahy and Iranshahi, 2011), and anti-fertility effect (Kalita et al., 2011). A large number of active compounds have been isolated from genus Ferula. Mainly sesquiterpene, coumarins, and sulfur containing compounds have been previously reported (Buddrus et al., 1985; Appendino et al., 1993; El-Razek et al., 2003; Bandyopadhyay et al., 2006).

Different substances like foods, pharmaceuticals or some cosmetic agents exhibited cytotoxic property. The main objective of cancer management with chemotherapy (anticancer drugs) is to kill the neoplastic (cancerous) cell instead of a normal healthy cell. Different types of cells present in human as part of immune system including natural killers, cytotoxic cells, and lymphokine activated cells are responsible to destroy abnormal and damaged cells (Cano et al., 2011). Any agent having cytotoxic activity can be used in various pathological conditions (inflammation, AIDS, infection, and cancer; Su et al., 2009). The current studies revealed the bioassay-guided isolation of two sesquiterpene coumarins and their anticancer potential against PC3 cells (prostate cancer).

## Materials and methods

### Plant material

Whole plant of *F. narthex* was collected from Chitral located in Khyber Pakhtunkhwa, Pakistan, in July 2010, and was identified by the Taxonomy section Department of Botany University of Peshawar. A voucher specimen (BOT 20002) was submitted to the herbarium section of the same department.

### Extraction and isolation of compounds

The collected plant of *F. narthex* was air dried in shade and was then powdered. Dry powder (8.0 kg) was extracted by methanol as an extraction solvent at room temperature for 14 days with daily shaking. After this it was filtered and the crude methanolic extract (900 g) was obtained which was concentrated under vacuum at low temperature (45°C). Then it was partitioned into n-hexane (70.0 g), chloroform (40.0 g), ethylacetate (29.0 g), and butanol (34.0 g) fractions. Chloroform fraction (40.0) was loaded onto silica gel column with increasing polarity of n-hexane/ethylacetate, which yielded number of sub-fractions A-G out of these fractions D (3.4 g) and E (1.3 g) obtained from 25 and 35% ethyl acetate/n-hexane respectively were resubjected to column chromatography. Sub-fraction D was further subjected to silica gel column by using n-hexane/acetone as eluting agent, which yielded compound 2 (600 mg) (8% acetone/n-hexane), While compound 1 (24.0 mg) was obtained from sub-fraction E at 9% acetone/n-hexane solvent system.

### Anticancer assay

Anticancer activity of compounds was evaluated in 96-well flat-bottomed micro plates by using the standard reduction of MTT colorimetric assay (Dimas et al., 1998). For this purpose, PC3 cells (prostate cancer) were cultured in DMEM, containing 5% of FBS, 100 μg mL^−1^ of streptomycin and 100 IU mL^−1^ of penicillin in 25 cm^3^ flasks, in an incubator at 37°C under a 5% carbon dioxide atmosphere. The exponentially growing cells were counted with a haemocytometer and diluted to a concentration of 1 × 10^5^ cells mL^−1^. The diluted culture was then introduced into 96-well plates (100 μL well^−1^) with various concentrations of the compounds in the range 1–100 μM and incubated overnight. After incubation, medium was separated and 50 μL MTT (2 mg mL^−1^) was added to each well and incubated further for 4 h. Subsequently, 100 μL of DMSO was added to each well. The MTT was reduced to formazan within viable cells and its absorbance was measured at 570 nm using a microplate ELISA reader (Spectra Max plus, Molecular Devices,CA, USA).

$$\begin{array}{l} \%inhibition=(\text{1}-Absorbance_{testcompound}/Absorbance_{control})\\ \;\times \text{100} \end{array}$$

### Molecular docking

The molecular docking procedure was widely used to predict the binding interaction of the compounds in the binding pocket of the enzyme. The 3D crystal structure of human histone acetyltransferase was downloaded from Protein Data Bank (PDB ID: 4PZS) (Oikonomakos et al., 2000). All the ions and water molecules were removed and the hydrogen atoms were added to the enzyme by the 3D protonation using the MOE (Molecular Operating Environment) (www.chemcomp.com) software. The target enzyme were then energy minimized by the default parameters of the MOE for the stability and further assessment of the enzyme. The structures of the compounds were built in MOE and energy minimized using the MMFF94x forcefield and gradient: 0.05. The synthesized compounds were docked into the active site of the target enzyme in MOE by the default parameters i.e., Placement: Triangle Matcher, Rescoring: London dG. For each ligand ten conformations were generated. The top-ranked conformation of each compound was used for further analysis.

### Statistical analysis

IC_50_ evaluation of only active compounds was done, serial dilutions of test compounds were prepared with different concentrations. A triplicate sample of each concentration was incubated using the standard procedure described. Percentage inhibition for each concentration was calculated. IC_50_ valve is evaluated using EZ-FIT, Enzyme kinetics software (Perrella Scientific, Inc., Hillsborough, NH, USA).

IC_50_-value was presented as mean ± *S.E.M* (standard error of the mean) calculated by using the formula

$$\begin{array}{l} S.E.M=\;\frac{s}{\sqrt{N}} \end{array}$$

where *s* = sample standard deviation

$$\begin{array}{l} s=\sqrt{\frac{1}{N-1}\overset{N}{\underset{i=1}{\sum }}{\left(x_{i}-\bar{x}\right)}^{2}} \end{array}$$

*x*_*i*_ −*x*_*n*_ = sample data set; $\bar{x}=$ mean valve of sample data; *N* = size of data.

## Results and discussion

### Identification of compounds

**Figure 1:**
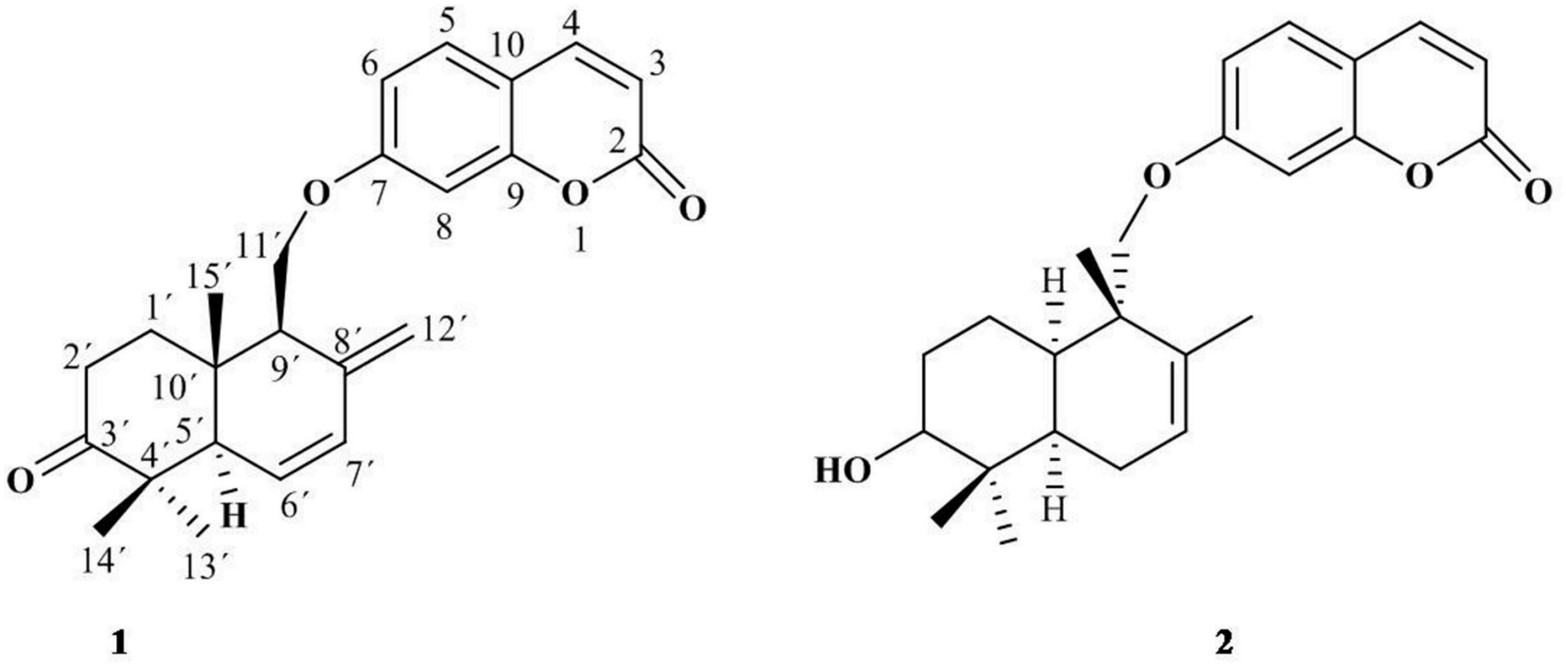
**Structures of compound 1 and 2**.

Compound 1 was isolated as a white amorphous solid from the chloroform-soluble part of the crude extract of *F. Narthex* Boiss. Its molecular formula C24H30O4 was deduced with the help of 13C NMR as well as HRESI-MS. Compound 1 (Figure 1) was found to be a sesquiterpene coumarin. The detail spectroscopic data of compound 1 were already reported in our previous article (Bashir et al., 2014). All of the spectral data of compound 2 have molecular formula C24H30O4 was found to be unambiguously matched with the reported data for conferol, previously isolated from *Ferula pallida* (Su et al., 2000).

### Anticancer activity

Natural organic compounds have extensively used as a new pharmacophore template in drug discovery. The bio-assay guided isolation of new anti-cancer agents (compounds **1** and **2**) were performed from *F. narthex*. In the preliminary step of bio-assay guided isolation the all crude fractions were evaluated for their anti-cancer activity against PC3 cells (prostate cancer). As a result, the *n*-hexane fraction showed significant activity against PC_3_ cancer lines with an IC_50_ value of 5.434 ± 0.249 μg/mL, followed by crude MeFn extract 7.317 ± 0.535 μg/mL, and CHCl_3_ fraction 9.613 ± 0.548 μg/mL (Table 1). After fractionation of chloroform, the compounds **1** and **2** were isolated. Both the isolated compounds were also evaluated against PC_3_ cancer lines by employing the same protocol that were used for the fractions Among tested pure compounds, only the compound **1** showed good anticancer activity with an IC_50_ value of 14.074 ± 0.414 μg/mL. The remaining fractions and compound **2** showed no activity against PC_3_cancer cell lines and the IC_50_ values of these were >30 as presented in Table 1.

**Table 1:** **Anticancer activity of crude MeFn extract and various fractions along with pure compounds 1 and 2**.

**S. No**.	**Sample**	**IC_50_ ± SD (μg ml^−1^)**
1	MeFn	7.317 ± 0.535
2	*n*-hexane	5.434 ± 0.249
3	CHCl_3_	9.613 ± 0.548
4	EtOAc	>30
5	BuOH	>30
6	Aqueous	>30
**ISOLATED COMPOUNDS**		
7	**1**	14.074 ± 0.414
8	**2**	>30
9	Doxorubicin (standard)	2.8 ± 0.12

As reported previously regarding the anticancer potential of *Ferula*, the oleo-gum resin of *F. foetida* was reported to prevent carcinogenesis (Saleem et al., 2001). This activity support the use of various isolated compounds from this genus for treatment of cancer along with other co-administered anticancer agents like vincristine (Unnikrishnan and Kuttan, 1990; Saleem et al., 2001). A prenylated coumarin (diversin, 1) together with four new sesquiterpene lactones (diversolides A, D, F, and G, 2-5) isolated from the roots of Ferula diversivittata were showed cancer chemo preventive activity (Iranshahi et al., 2010). Galbanic acid isolated from F. assafoetida was reported to exhibit anticancer effect (Kim et al., 2011).

### Molecular docking simulation

**Figure 2:**
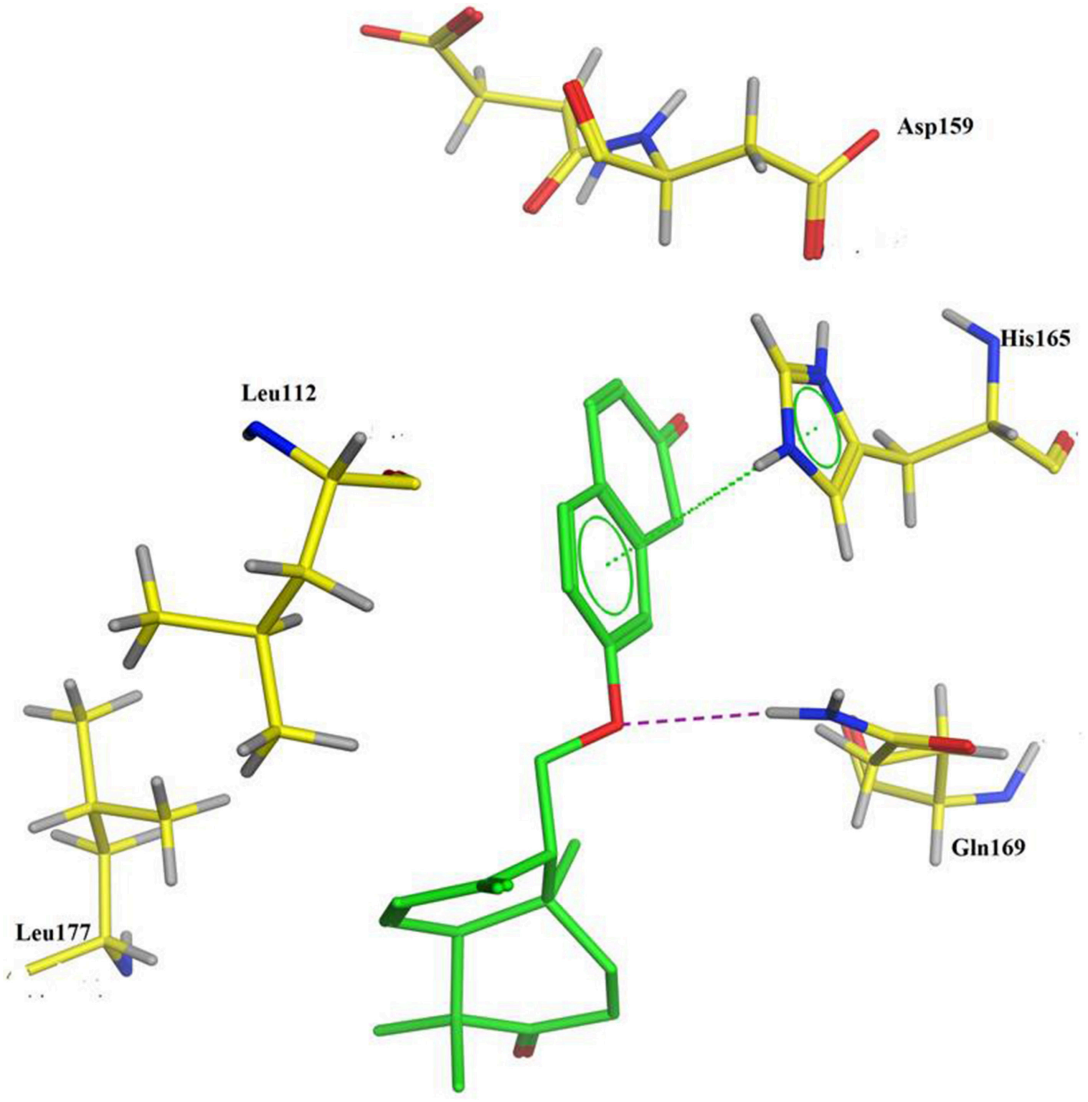
**Docking conformation of compound 1 (generated by MOE docking software) in the active site of human histone acetyltransferase enzyme**.

PASS (Prediction of Activity Spectra) is an online tool (Lagunin et al., 2000), which predicts almost 900 types of activities based on the structure of a compound. PASS analysis (Table 2) of the compound **1** predicts anti-cancer activity (antineoplastic) with Pa (probability to be active) value of 0.303. To evaluate the inhibitory nature of the compound **1** as anti-cancer agent, the compound **1** was docked against human histone acetyltransferase (anti-cancer drug target), molecular docking simulations were carried out. Molecular docking is an efficient method to get an insight into ligand-receptor interactions. Molecular docking studies were performed using Molecular Operating Environment (MOE) software (www.chemcomp.com). The 3D crystal structure of human histone acetyltransferase was downloaded from Protein Data Bank (PDB ID: 4PZS) (Oikonomakos et al., 2000). Before docking experiment, the structure of compound **1** was prepared for docking by minimizing its energy using MOE. Most macromolecular crystal structures contain little or no hydrogen coordinate data due to limited resolution and thus protonation was done prior to docking using Protonate 3D tools implemented in MOE. Protonation was followed by energy minimization up to 0.05 Gradient using Amber99 forcefield. The docking protocol predicted the same conformation as was present in the crystal structure with RMSD value close to the allowed range (Paul and Mukhopadhyay, 2004) and surrounded by the same active site residues of the enzyme. Amongst the generated docking conformations the top-ranked conformation was visualized for ligand-enzyme interaction using MOE. Analysis of the docking results showed that compound **1** fit well within the active site of histone acetyltransferase enzyme (Figure 2). From the docking conformation it was observed that oxygen atom of the compound established hydrogen bond with the active site residue Gln169. The phenyl ring of the compound formed arene-arene interaction with the active site residue His165 (Figure 2). Furthermore, several hydrophobic interactions between compound **1** and the hydrophobic residues of the enzyme were also observed. The presence of hydrogen bond, arene-arene and hydrophobic interactions confirm that the compound **1** may be specific to this site. These preliminary results suggest that the compound **1** might exhibit inhibitory activity against histone acetyltransferase enzyme. These computational predictions are verified by our experimental results in the compound **1** showed good anti-cancer activity (Table 1).

**Table 2:** **PASS prediction of the compound, Pa represents probability to be active and Pi represents probability to be inactive**.

**Pa**	**Pi**	**Predicted activity**
0.288	0.017	UGT2B9 substrate
0.330	0.062	Antipsoriatic
0.266	0.007	Testosterone agonist
0.293	0.034	UGT2B17 substrate
0.351	0.093	CYP3A5 substrate
0.273	0.016	UGT2B18 substrate
0.287	0.031	UGT2B substrate
0.304	0.051	UDP-glucuronosyltransferase substrate
0.275	0.027	Cholesterol synthesis inhibitor
**0.303**	**0.056**	**Antineoplastic (pancreatic cancer)**
0.261	0.017	Menstruation disorders treatment
0.294	0.051	UGT1A substrate
0.280	0.040	Choleretic
0.362	0.122	Cytoprotectant
0.271	0.032	ATP-binding cassette A1 stimulant
0.293	0.060	Indanol dehydrogenase inhibitor
0.326	0.095	Analeptic
0.238	0.011	Hair growth stimulant
0.306	0.079	Antidiabetic
0.287	0.061	NF-E2-related factor 2 stimulant

The compound ***1*** predicts anti-cancer activity with Antineoplastic (pancreatic cancer).

## Conclusion

The anticancer activity showed that *F. narthex* can be used in the management of cancer as *n*-hexane fraction, crude MeFn, CHCl_3_ fraction, and pure compound **1** showed good to moderate anticancer activity against PC_3_ cancer lines. Moreover the activity support the use of different compounds isolated from the genus *Ferula* as anticancer agents.

### Mathematical formula

Standard deviation

$$\begin{array}{l} s=\sqrt{\frac{1}{N-1}\overset{N}{\underset{i=1}{\sum }}{\left(x_{i}-\bar{x}\right)}^{2}} \end{array}$$

x_i_ − x_*n*_ = sample data set$;;\;\bar{\text{x}}=$ mean valve of sample data; *N* = size of data.

## Author contributions

BA and ARK gave the project idea. Ajmal Khan and SB were project supervisors. MA performed isolation of compounds and AR helped in structure elucidation. Ayesha Khan and AA performed anticancer activity. AW and AKJ carried out docking studies. Ajmal Khan was also involved in the useful discussion and participated in manuscript writing. All authors read and approved the final manuscript.

### Conflict of interest statement

The authors declare that the research was conducted in the absence of any commercial or financial relationships that could be construed as a potential conflict of interest.

## Abbreviations

EtoAc: ethylacetate
MeFn: methanolic extract of *Ferula narthex* Bioss
MTT, (3-(4, 5-dimethylthiazol-2-yl)-2: 5-diphenyltetrazolium bromide)
DMEM: dulbecco-modified eagle medium
FBS: fetal bovine serum
DMSO: dimethyl sulfoxide
IC_50_: 50% inhibitory concentration
SD: standard deviation
NMR: nuclear magnetic resonance
HRESI-MS: high-resolution electrospray ionization mass spectrometry.
