# Corrigendum: Bioassay-Guided Isolation of Sesquiterpene Coumarins from *Ferula narthex* Bioss: A New Anticancer Agent

**DOI:** 10.3389/fphar.2016.00444

**Published:** 2016-11-22

**Authors:** Mahboob Alam, Ajmal Khan, Abdul Wadood, Ayesha Khan, Shumaila Bashir, Akhtar Aman, Abdul Khaliq Jan, Abdur Rauf, Bashir Ahmad, Abdur Rahman Khan, Umar Farooq

**Affiliations:** ^1^Department of Pharmacy, Abbottabad University of Science and Technology HavelianAbbottabad, Pakistan; ^2^Department of Chemistry, COMSATS Institute of Information TechnologyAbbottabad, Pakistan; ^3^Department of Biochemistry, Abdul Wali Khan University-MardanMardan, Pakistan; ^4^Department of Pharmacy, University of PeshawarPeshawar, Pakistan; ^5^Department of Pharmacy, Shaheed Benazir Bhutto University SheringalSheringal, Pakistan; ^6^Department of Chemistry, Shaheed Benazir Bhutto University SheringalSheringal, Pakistan; ^7^Department of Chemistry, University of SwabiKhyber Pakhtunkhwa, Pakistan; ^8^Centre of Biotechnology and Microbiology, University of PeshawarKhyber Pakhtunkhwa, Pakistan

**Keywords:** *Ferula narthex* Bioss, sesquiterpene coumarin, cytotoxic, anticancer potential, prediction of activity spectra, human histone acetyltransferase, and molecular docking

Due to an oversight, there was an error in Section “Results and Discussion,” Sub-section “Identification of Compounds,” in paragraph one. It should read: “Compound 1 was isolated as a white amorphous solid from the chloroform-soluble part of the crude extract of *F. Narthex* Boiss. Its molecular formula C_24_H_30_O_4_ was deduced with the help of ^13^C NMR as well as HRESI-MS. Compound 1 (**Figure 1**) was found to be a sesquiterpene coumarin.

Compound 2 (**Figure 1**) was also found to be a sesquiterpene coumarin based on characteristic peaks in the ^1^H and ^13^C NMR spectra. The detail spectroscopic data of compound 1 and compound 2 were already reported in our previous article (Bashir et al., 2014).

The authors would like to apologize for any inconvenience caused and this change does not affect the scientific conclusions of the article in any way.

## Conflict of interest statement

The authors declare that the research was conducted in the absence of any commercial or financial relationships that could be construed as a potential conflict of interest.
